# Data-Driven Self-Triggered Control for Networked Motor Control Systems Using RNNs and Pre-Training: A Hierarchical Reinforcement Learning Framework

**DOI:** 10.3390/s24061986

**Published:** 2024-03-20

**Authors:** Wei Chen, Haiying Wan, Xiaoli Luan, Fei Liu

**Affiliations:** Key Laboratory of Advanced Process Control for Light Industry, Institute of Automation, Jiangnan University, Wuxi 214122, China; wchen5600@gmail.com (W.C.); haiyingwan96@gmail.com (H.W.); fliu@jiangnan.edu.cn (F.L.)

**Keywords:** self-triggered control, recurrent neural networks, pre-training, hierarchical reinforcement learning, networked motor control systems

## Abstract

This paper introduces a novel data-driven self-triggered control approach based on a hierarchical reinforcement learning framework in networked motor control systems. This approach divides the self-triggered control policy into higher and lower layers, with the higher-level policy guiding the lower-level policy in decision-making, thereby reducing the exploration space of the lower-level policy and improving the efficiency of the learning process. The data-driven framework integrates with the dual-actor critic algorithm, using two interconnected neural networks to approximate the hierarchical policies. In this framework, we use recurrent neural networks as the network architecture for the critic, utilizing the temporal dynamics of recurrent neural networks to better capture the dependencies between costs, thus enhancing the critic network’s efficiency and accuracy in approximating the multi-time cumulative cost function. Additionally, we have developed a pre-training method for the control policy networks to further improve learning efficiency. The effectiveness of our proposed method is validated through a series of numerical simulations.

## 1. Introduction

In various industrial settings, direct current (DC) motors are critical as actuators, performing key production tasks. However, these motors are often numerous, making it difficult to uniformly supervise them during production. As a result, networked motor control systems (NMCS) have emerged as a solution [1]. In NMCS, motor control systems are connected to a control center via a communication network, which can significantly aid factories in cutting down on expenses and boosting production efficiency. Nonetheless, these systems also pose certain challenges: the large number of control subsystems significantly increases the demand for computational and communication resources. To address these challenges, self-triggered control offers a promising solution. It strategically schedules communications and computations, reducing network load and processor usage [2,3].

Self-triggered control systems grant control subsystems the autonomy to decide their own schedule for data recalculation and transmission [4,5]. This decision-making process is based on the real-time status of the system and predefined performance goals. These self-triggered control methods, drawing from input-to-state stability (ISS), instigate control updates contingent on the progression of the system’s state, thereby ensuring stability while optimizing performance. ISS takes into account the system’s inputs and the evolution of its states, establishing a relationship between them to determine the system’s resilience to external disturbances. Specifically, if a system is considered ISS, it exhibits a certain tolerance to input perturbations, ensuring that it does not lose stability due to disturbances [6,7,8]. Then, minimum attention is another approach for implementing self-triggered control, aimed at optimizing the timing of control input updates. The concept of minimum attention ensures that control input updates do not occur too frequently, reducing computational resource consumption and improving system efficiency. By determining the shortest time interval between two consecutive control input updates, minimum attention helps strike a balance between system performance and resource consumption [9,10]. These model-based self-triggered control methods have demonstrated their effectiveness in industrial environments, effectively minimizing the use of computational resources [11,12,13].

Although model-based self-triggered control brings many benefits to industrial applications, it also faces several challenges. A major issue is that model-based self-triggered control strategies depend heavily on accurate models of the plant. However, real-world systems frequently experience uncertainties, varying parameters, and environmental shifts [14]. Moreover, within the context of NMCS, creating models for thousands of control subsystems can be expensive. In response to these challenges, the concept of data-driven self-triggered control emerges. These approaches represent a model-free control methodology that negates the necessity for plant-specific model information. Instead, it solely depends on the input-output data stored in the system to formulate the controller [15,16].

As a data-driven method, reinforcement learning has been frequently used in recent years to design model-free, self-triggered controllers. It is a machine learning approach where an algorithm learns to make decisions by interacting with an environment, using trial and error to discover the best strategy. In the context of model-free, self-triggered control based on reinforcement learning, the controller continuously adjusts its strategy based on the feedback costs from its interaction with the plant, thereby developing a self-triggered control strategy that generates both control actions and triggering intervals [17,18,19]. However, these previous studies often focused on designing appropriate rewards for self-triggered control systems, neglecting the hierarchical structure of the self-triggered control strategies. This neglect has led to increased complexity in learning self-triggered control strategies, consequently reducing the efficiency of the learning process. Therefore, considering the hierarchical structure of strategies is crucial to model-free, self-triggered control based on reinforcement learning. We propose adopting the concept of hierarchical reinforcement learning (HRL), which divides self-triggered control into higher-level and lower-level strategies, allowing each layer to have its own distinct tasks and optimization objectives. This is our primary research motivation [20,21,22].

Furthermore, in implementing HRL, neural networks are often utilized to approximate control policies and value functions. Compared to traditional neural networks, recurrent neural networks (RNNs) provide memory capabilities that enhance data representation and comprehension. It overcomes the constraints of feedforward networks, extending their utility to tasks like time-series forecasting. Similarly, self-triggered control, being time-dependent and linking current to past states, benefits from RNNs’ superior time-step dependency modeling. Therefore, how to use recurrent neural networks to better capture the temporal characteristics in self-triggered control systems becomes an important issue, which constitutes our second research motivation [23,24].

Additionally, in HRL, the initial parameters of neural networks have a significant impact on an agent’s learning and performance, especially when target states are difficult to reach. In scenarios where reinforcement learning is applied to control problems, the choice of an initial policy can affect whether the agent successfully learns an effective strategy. To address these issues, pre-training can kick-start the learning process by providing the model with a foundational understanding before it begins learning from environmental interaction. It also garners prior knowledge by mastering a control strategy conducive to system convergence, thereby formulating a versatile approach for self-triggered control. Therefore, it is necessary to pre-train the policy neural network before formally entering the reinforcement learning stage, which is the third research motivation [25,26].

Based on the above content, this paper presents a novel approach to model-free, self-triggered control based on HRL for NMCS. This framework segments the self-triggered control strategy into higher and lower layers, where the higher layer guides the lower layer in decision-making, thereby reducing the exploration space of the lower layer and enhancing the efficiency of the reinforcement learning process. On the other hand, RNNs are utilized in developing a dual-actor critic algorithm within the HRL framework for efficient model-free self-triggered control in order to precisely approximate the Q-function and improve the overall cost-efficiency and model accuracy of the control process. Lastly, to further enhance the efficiency of the reinforcement learning process, we propose a pre-training method for the data-driven self-triggered controller in NMCS. Our method allows for pre-training of untrained networks based on previously trained neural networks, enabling the policy networks to acquire prior knowledge about self-triggered control before entering the reinforcement learning stage, thereby accelerating the subsequent reinforcement learning process. The main contributions of this research are:

- Introduction of a novel model-free self-triggered control framework that employs a hierarchical strategy to reduce the exploration space for the lower-level policy under the guidance of the higher-level strategy. This approach significantly reduces computational burdens in Networked Motor Control Systems (NMCS) and enhances the efficiency of the learning process.
- The strategic employment of RNNs empowers the critic network to adeptly capture the temporal dynamics inherent in the learning phase, markedly improving the precision and stability of the critic network in approximating the multi-time cumulative cost function.
- Implementation of a novel pre-training methodology that endows the control strategy sets the stage for the system to achieve convergence from its initial state, thereby bolstering training efficiency.

The remainder of this paper is structured as follows: Section 2 provides an overview of NMCS. Section 3 offers an initial examination of the hierarchical model-free self-triggered controller design. In Section 4, the utilization of the dual-actor critic algorithm based on RNNs for optimizing the self-triggered control policy is proposed. Section 5 introduces the pre-training method based on a previously trained network. Section 6 illustrates the effectiveness of the proposed method through numerical examples. Finally, Section 7 concludes the paper with a summary of the results.

## 2. Networked Motor Control Systems

**Figure 1:**
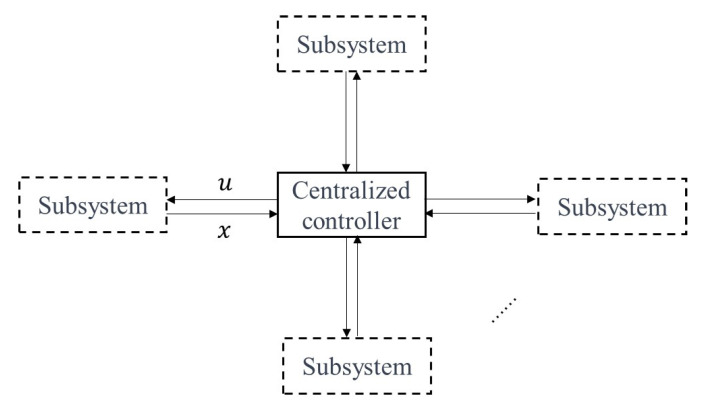
Networked motor control systems.

The NMCS comprises *N* interconnected motor subsystems, as illustrated in Figure 1. At the heart of the system lies a central controller, serving as the pivotal decision-maker. This controller assesses the collective status and objectives of the system and subsequently dispatches commands to each component or device. Leveraging sophisticated communication networks, the controller interfaces with the diverse subsystems. This arrangement empowers the central controller to efficiently orchestrate the operations of the entire network, ensuring optimal performance and seamless integration across all motor subsystems.

Each subsystem can be regarded as an independent motor control system, which unfolds into five key components: power supply, motor, sensors, controller, and actuators. The power supply provides the needed electrical energy to the motor, which then turns this electricity into mechanical power for use. Sensors are strategically placed to carefully track the motor’s performance and the conditions around it. After getting feedback from the sensors, the controller focuses on creating control strategies and then sends control signals to the actuators. Then, the actuators, following these instructions, start actions that match the control signals.

As a commonly utilized electric motor in industrial production, the DC brushed motor includes essential components such as the stator, rotor (or armature), brushes, and a commutator. It transforms electrical energy into mechanical rotation by harnessing the interplay of magnetic fields. The stator produces a constant magnetic field around the rotor. When electric current flows through the rotor windings, it generates a secondary magnetic field. The interaction between these magnetic fields creates torque, prompting the rotor to spin. The brushes sustain an electrical connection with the rotating rotor and the motor’s stationary elements, while the commutator cyclically reverses the flow of current to maintain consistent rotational movement.

**Figure 2:**
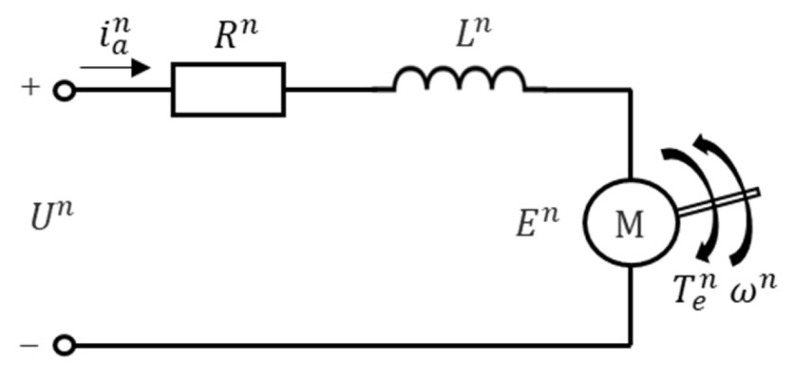
The equivalent circuit diagram of the Brushed DC motor.

For each subsystem *n*, the equivalent circuit diagram of the brushed DC motor is as shown in Figure 2. In this context, the meaning of each parameter in the block diagram is given as follows:

- $U^{n}$ represents the voltage applied across the armature of subsystem *n*, respectively.
- $R^{n}$ is the armature resistance of subsystem *n*, respectively.
- $L^{n}$ denotes the armature inductance of subsystem *n*, respectively.
- ${i_{a}}^{n}$ is the current flowing through the motor armature of subsystem *n*, respectively.
- $E^{n}$ stands for the counter-electromotive force generated by cutting the magnetic field in the armature of subsystem *n*, respectively.
- $\omega^{n}$ represents the angular velocity of the motor shaft of subsystem *n*, respectively.
- ${T_{e}}^{n}$ represents the electromagnetic torque of the motor shaft of subsystem *n*, respectively.

Then, according to Kirchhoff’s voltage law, the electrical equation representing a motor’s input circuit describes the relationship between voltage, current, resistance, and back electromotive force (EMF). This relationship is given by:

(1) $$U^{n}=R^{n}{i_{a}}^{n}+L^{n}\frac{d{i_{a}}^{n}}{dt}+E^{n}.$$

During motor operation, the relative motion between the magnetic field and the armature coil induces an EMF within the coil. This induction is based on Faraday’s law of electromagnetic induction, expressed as:

(2) $$E^{n}={k_{e}}^{n}{\phi_{f}}^{n}\omega^{n},$$

where ${k_{e}}^{n}$ is the back electromotive force constant, and ${\phi_{f}}^{n}$ is the magnetic flux constant.

In a similar vein, a direct current motor’s armature current-carrying conductor, when influenced by the stator magnetic field, generates an electromagnetic torque. This torque is described by the equation,

(3) $${T_{e}}^{n}={k_{t}}^{n}\cdot {\phi_{f}}^{n}\cdot {i_{a}}^{n},$$

where ${k_{t}}^{n}$ is the torque constant.

Furthermore, the mechanical load equation delineates the relationship between the mechanical load applied to a motor and the motor’s resulting motion or behavior.

(4) $${T_{e}}^{n}=J^{n}\cdot \frac{d\omega^{n}}{dt}+{B_{v}}^{n}\cdot \omega^{n},$$

where $J^{n}$ is the moment of inertia of the load constant and ${B_{v}}^{n}$ is the viscous damping coefficient.

Finally, by considering voltage $u^{n}$ as the input and defining the state variable as $x^{n}={\left[{i_{a}}^{n}\;\omega^{n}\right]}^{T}$, we can derive the continuous state-space equation for the brushed DC motor control system as follows.

(5) $${\dot{x}}^{n}=\left[\begin{array}{cc} -\frac{R^{n}}{L^{n}} & -\frac{{k_{e}}^{n}}{L^{n}}\\ \frac{{k_{t}}^{n}}{J^{n}} & -\frac{{B_{v}}^{n}}{J^{n}} \end{array}\right]x^{n}+\left[\begin{array}{c} \frac{1}{L^{n}}\\ 0 \end{array}\right]u^{n}=A^{n}x^{n}+B^{n}u^{n}.$$

Since NMCS usually uses digital computers as its operating platform, we need to convert the continuous state-space model into a discrete state-space model. Assuming that the discrete time is dt, the discrete state-space model is:

(6) $$x_{t+1}^{n}=\left[\left[\begin{array}{cc} -\frac{R^{n}}{L^{n}} & -\frac{{k_{e}}^{n}}{L^{n}}\\ \frac{{k_{t}}^{n}}{J^{n}} & -\frac{{B_{v}}^{n}}{J^{n}} \end{array}\right]\cdot dt+I\right]x_{t}^{n}+\left[\left[\begin{array}{c} \frac{1}{L^{n}}\\ 0 \end{array}\right]\cdot dt\right]u_{t}^{n}=A_{d}^{n}x_{t}^{n}+B_{d}^{n}u_{t}^{n}.$$

In this manner, we can employ the discrete linear state-space equation for the subsystem of NMCS to design the controller. However, there still exist challenges to be addressed here. Within NMCS, bandwidth limitation emerges as a significant issue, primarily pertaining to the network’s communication capability. When the network’s communication demands exceed its capability, a bandwidth limitation occurs. Therefore, employing self-triggered mechanisms to overcome the network bandwidth limitation issue in NMCS holds significant meaning.

## 3. Model-Free Self-Triggered Control Based on Hierarchical Reinforcement Learning

In this section, we explore an innovative methodology for NMCS: a data-driven, model-free, self-triggered control strategy that employs hierarchical reinforcement learning. This cutting-edge approach aims to minimize the consumption of system computational resources. Central to our discussion is the control of a linear time-invariant (LTI) system, as described by Equation (6). The controller in this system engages with the plant at predetermined moments, each separated by a triggering time interval $\tau_{t}$—signifies the duration between consecutive triggering instants, as defined by the triggering strategy H. In parallel, the control strategy U utilizes the system state $x_{t_{l}}$ to achieve predetermined performance objectives. For each control interval, the strategy generates control commands ${\bar{u}}_{l}$ derived from the current system state $x_{t_{l}}$. These commands remain constant over the interval $\tau_{t}$ up until the next triggering instant $t_{l+1}$, when they are recalculated. This process ensures a specified level of control performance for the plant. Mathematically, it is only when,

(7) $$\left\{\begin{array}{c} t_{l+1}=t_{l}+H\left(x_{t_{l}}\right)=t_{l}+\tau_{t}\\ \left.u_{t}={\bar{u}}_{l}=U\left(x_{t_{l}}\right),\;t\in N_{\left[t_{l},t_{l+1}\right)}\right. \end{array}\right.$$

The fundamental objective of self-triggered control is to strategically minimize the frequency of input updates. This is achieved by maximizing the duration of the triggering time interval $\tau_{t}$ while still ensuring that the system maintains a predetermined level of performance. To assess the performance of the self-triggered control strategy, as described by Equation (7), we employ a performance indicator given by:

(8) $$J=\sum_{t=t_{l}}^{\infty }\left(x_{t}^{T}Qx_{t}+u_{t}^{T}Ru_{t}\right),$$

In this equation, *Q* and *R* are positive definite matrices, serving as weighting matrices.

When considering the triggering moment $t_{l}$ as the initial time instant and acknowledging that the control input $u_{t}={\bar{u}}_{l}$ remains constant within the triggering time interval $\tau_{t}$, the performance evaluation metric at $t_{l}$ can be formulated as:

(9) $$J=\sum_{t=t_{l}}^{t_{l}+\tau_{t}}\left(x_{t}^{T}Qx_{t}+{\bar{u}}_{l}^{T}R{\bar{u}}_{l}\right)+\sum_{t=t_{l+1}}^{\infty }\left(x_{t}^{T}Qx_{t}+u_{t}^{T}Ru_{t}\right).$$

In the context of self-triggered linear quadratic control, as proposed by T. Gommans (2014) [27], we utilize a Lyapunov function $V_{\beta }\left(x\right)=\beta x^{T}Px$, where *P* represents the unique positive semi-definite solution to the discrete algebraic Riccati equation (DARE), and β≥1 acts as scaling factors. This function approximates the performance index from $t_{l+1}$ to infinity, leading to a revised performance index,

(10) $$J=\sum_{t=t_{l}}^{t_{l}+\tau_{t}}\left(x_{t}^{T}Qx_{t}+{\bar{u}}_{l}^{T}R{\bar{u}}_{l}\right)+V_{\beta }\left(x_{t_{l+1}}\right).$$

Similarly, the stability of the system is analyzed using the Lyapunov function method, which provides a triggering condition,

(11) $$\sum_{t=t_{l}}^{t_{l+1}+\tau_{t}}\left(x_{t}^{T}Qx_{t}+{\bar{u}}_{l}^{T}R{\bar{u}}_{l}\right)+V_{\beta }\left(x_{t_{l+1}}\right)\leq V_{\beta }\left(x_{t_{l}}\right).$$

The left side of the inequality (11) reflects the performance metric of the system under the effect of the self-triggered control strategy. Additionally, the future cumulative cost is approximated by employing the Lyapunov function associated with the state, thereby converting the global control performance metric into the form of Expression (10). Thus, the triggering condition suggests that, within the self-triggered control strategy, the control performance must be limited by the Lyapunov function to guarantee the system’s convergence. This setup allows for a degree of sub-optimal control performance, which corresponds to a percentage of the periodic control performance. By adjusting the value of β, a balance can be struck between control performance and computational cost. Under the constraints (11), the current state $x_{t}$ and control variable ${\bar{u}}_{l}$ determine whether maintaining ${\bar{u}}_{l}$ constant over $\tau_{t}=t_{l+1}-t_{l}$ will lead to stable system convergence. The control strategy U prescribes the sub-optimal control variable ${\bar{u}}_{l}$, capable of minimizing the performance index (10), while the triggering strategy H aims to maximize the triggering time interval, adhering to the triggering condition. The self-triggered control strategy thus reformulates:

(12) $$\left\{\begin{array}{c} H\left(x_{t_{l}}\right)=\max\left\{\tau_{t}|\sum_{t=t_{l}}^{t_{t+1}+\tau }\left(x_{t}^{T}Qx_{t}+{\bar{u}}_{l}^{T}R{\bar{u}}_{l}\right)+V_{\beta }\left(x_{t_{l+1}}\right)\leq V_{\beta }\left(x_{t_{l}}\right)\right\}\\ U\left(x_{t_{l}},\tau_{t}\right)=\underset{{\bar{u}}_{l}}{\arg\min}\left\{\sum_{t=t_{l}}^{t_{t+1}+\tau_{t}}\left(x_{t}^{T}Qx_{t}+{\bar{u}}_{l}^{T}R{\bar{u}}_{l}\right)+V_{\beta }\left(x_{t_{l+1}}\right)\right\} \end{array}\right.$$

But the self-triggered methods previously mentioned rely heavily on an accurate system model, which often leads to substantial modeling costs. Our interest lies in developing a self-triggered controller that operates without a model. To address this challenge, we consider the use of HRL techniques for data-driven, self-triggered control.

Contrasting with traditional reinforcement learning, HRL is based on semi-markov decision processes (SMDP) and allows for decision-making across various time scales. This characteristic aligns well with the framework of self-triggered control, where the triggering strategy is crafted to ensure system stability and maximize the triggering interval $\tau_{t}$. Within these intervals, the control input ${\bar{u}}_{l}$ remains constant, as determined by the control strategy. Given that the control action updates only after each triggering interval, self-triggered control can be viewed as a type of SMDP with a non-fixed decision-making timescale.

**Figure 3:**
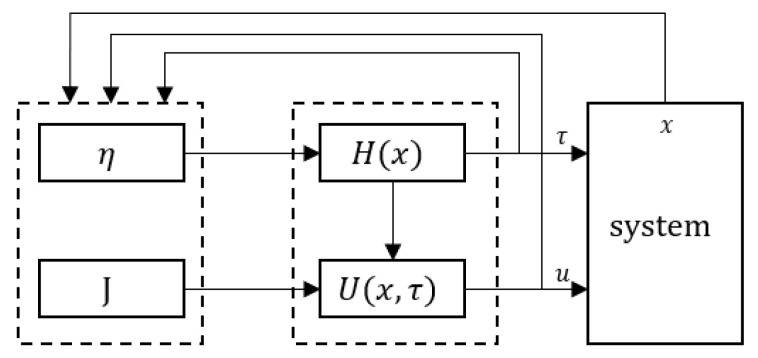
Hierarchical structure of self-triggered control.

Building on the principles of HRL, we partition the self-triggered control strategy into higher- and lower-level policies. The higher-level policy guides the lower-level policy in decision-making, effectively narrowing the search space for lower-level actions and enhancing the efficiency of the learning process [28,29,30]. Furthermore, organizing policies into layers allows each tier to pursue its own distinct tasks and optimization objectives, thereby increasing the clarity and interpretability of reinforcement learning results. Within the framework of model-free self-triggered control employing HRL, the triggering policy is the higher-level policy, whereas the control policy is implemented at the lower-level. Initially, the triggering time interval is established based on specific triggering conditions under a predefined initial policy, guaranteeing system stability (as shown in Figure 3).

Here, *J* and η serve as evaluation metrics for the triggering and control strategies, respectively, guiding the optimization of these policies. This essentially constitutes an iterative design process. To implement model-free self-triggered control, we consider employing the policy gradient method from reinforcement learning to derive the gradients of the parameters for both control and triggering strategies with respect to the two performance evaluation metrics, thereby guiding the iterative optimization of both strategies.

The subsequent question is how to design the evaluation metrics for the triggering and control strategies. We can observe that both the performance metric (10) and the triggering conditions (11) involve the cumulative cost within the triggering time interval, so we define the multi-time cumulative cost function,

(13) $$G\left(x_{t},{\bar{u}}_{l},\tau_{t}\right)=\sum_{t=t_{l}}^{t_{l}+\tau_{t}}\left(x_{t}^{T}Qx_{t}+{\bar{u}}_{l}^{T}R{\bar{u}}_{l}\right).$$

**Remark** **1.** *The multi-time cumulative cost function reflects the total cost accrued by the agent when continuously applying the control input ${\bar{u}}_{l}$ over the triggering time interval, starting from the initial state. This indicates a heightened focus on the local control performance from the initial time to the subsequent triggering time. In the optimization process, the triggering policy is initially optimized based on an initial control policy assessed through the triggering policy evaluation metric, which relies on the multi-time cumulative cost function. Subsequently, the control policy is refined under the optimized triggering policy, guided by the control policy evaluation metric, which is also dependent on the multi-time cumulative cost function. This procedure is iterated in subsequent cycles, facilitating a progressive iterative design of the self-triggered control strategy.*

Consequently, the formulation (10) is modified to:

(14) $$J=G\left(x_{t},{\bar{u}}_{l},\tau_{t}\right)+V_{\beta }\left(x_{\tau_{t}}\right).$$

Equation (14) serves as the performance evaluation metric for the self-triggered control strategy, highlighting the impact of the continuous control input ${\bar{u}}_{l}$ within the triggering interval $\tau_{t}$ at the current moment. Moreover, our aim is to achieve the longest possible triggering interval while satisfying the stability condition, thereby reducing computational resource consumption. In conjunction with the multi-time cumulative cost function, the stability triggering condition is reformulated as:

(15) $$G\left(x_{t},{\bar{u}}_{l},\tau_{t}\right)+V_{\beta }\left(x_{\tau_{t}}\right)\leq V_{\beta }\left(x_{0}\right).$$

The computational cost evaluation metric is given by:

(16) $$\eta ={\left(G\left(x_{t},{\bar{u}}_{l},\tau_{t}\right)+V_{\beta }\left(x_{\tau_{t}}\right)-V_{\beta }\left(x_{0}\right)\right)}^{2}.$$

**Remark** **2.** *Equation (16) is capable of assessing whether a given time interval satisfies the stability conditions, given the system state and control variables. If the left-hand side is less than the right-hand side, it indicates that the provided trigger time interval is overly conservative. Conversely, if the left-hand side is greater than the right-hand side, it would compromise the stability conditions. Therefore, the ideal scenario is to make the left-hand side as equal to the right-hand side as possible. In other words, the closer the value of η approaches zero, the better we consider the obtained trigger time interval to be.*

Now, we have two metrics *J*(14) and η(16) for evaluating control and triggering strategies. The next question is how we use them to guide policy optimization. With two hierarchical strategies in place, including the control strategy π and triggering strategy τ, along with corresponding strategy evaluation indices *J*(14) and η(16). We apply the gradient descent method. This involves computing the gradient of the strategy evaluation indices with respect to the strategy parameters and optimizing these parameters in the direction of the gradient descent. This process leads to the gradual optimization of the control and triggering strategies:

(17) $$\left\{\begin{array}{c} H_{i+1}=H_{i}-\nabla_{\tau }\eta \\ U_{i+1}=U_{i}-\nabla_{\pi }J \end{array}\right.$$

As the iteration number *i* increases, both the triggering and control strategies will be continuously optimized until the two performance metrics *J* and η converge, thereby achieving the iterative design process of model-free self-triggered control. In a model-free environment, a hierarchical self-triggered controller can be designed to ensure system stability and achieve sub-optimal control performance. The subsequent chapter will delve into the implementation of this gradient-descent-based optimization process through the dual-actor critic algorithm.

## 4. Dual-Actor Critic Algorithm Implementation Using Recurrent Neural Networks

For the hierarchical structure of self-triggered control mentioned in Figure 3, our aim is to optimize the initial policy to convergence using the dual-actor critic algorithm. The critic network fits the multi-time cumulative cost function (referenced in Equation (13)) and calculates two evaluation metrics to update the self-triggered control policy. The multi-time cumulative cost estimated by Equation (13) is strongly correlated with the system state variable *x* constrained by the system dynamics $\dot{x}=f\left(x,u\right)$. This implies a temporal dependence in the system state at each time step.

**Figure 4:**
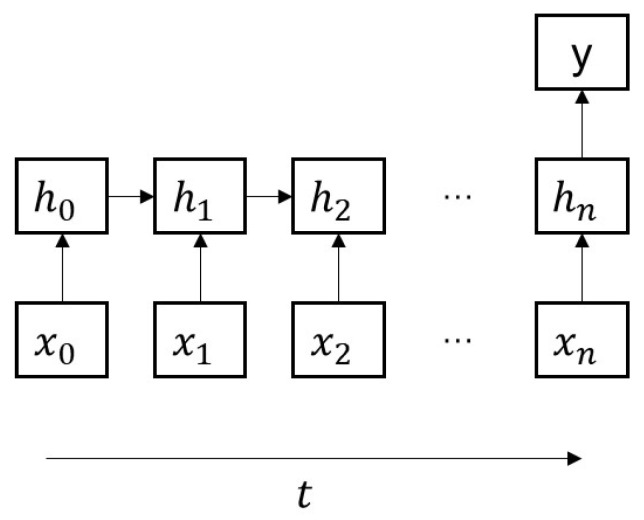
Input-output paradigm of RNNs.

**Figure 5:**
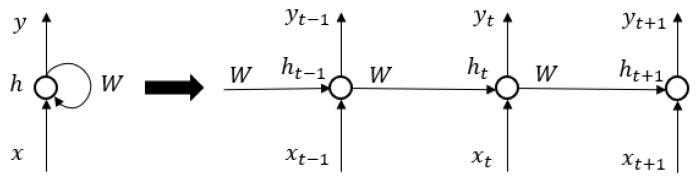
Parameter sharing in RNNs.

To capture the temporal dependence relationship, we employ RNNs, which are more suitable for processing time series data, to fit the multi-time cumulative cost function. RNNs, with their ability to maintain a memory of past states through hidden states, are well-suited to handle temporal dependencies in sequential data. This makes them effective for learning the multi-time cumulative cost function. Engineered to process sequences, RNNs adeptly manage both input and output sequences, setting them apart from networks tailored to fixed-sized inputs and outputs. In Figure 4, the representation $\left[x_{0},x_{1},\ldots ,x_{n}\right]$ symbolizes the input sequence over different time instances. In addition, RNNs share parameters across these instances, a unique feature that helps maintain consistency in processing sequential data and capturing temporal dependencies (as shown in Figure 5). This feature is critical for understanding and modeling the dynamics of control processes, which typically depend on past states and inputs.

The following equation depicts the input-output mapping of RNNs. The current hidden layer node $h_{t}$ is linked not only to the current input $x_{t}$ but also to the previous hidden layer node $h_{t-1}$:

(18) $$\begin{array}{c} h_{t}=\tanh\left(W_{xh}x_{t}+W_{hh}h_{t-1}+b_{h}\right)\\ y_{t}=W_{hy}h_{t}+b_{y}, \end{array}$$

where $W_{xh},W_{hh},W_{hy}$ denote the parameter matrices and $b_{h},b_{y}$ are the output biases. This feedback linkage endows RNNs with the capacity to capture and process temporal dependencies, primarily facilitated by their internal recurrent connections.

In the self-triggered control scheme, the system’s state is influenced by past events and control update timings, introducing delays and asynchronous behaviors. The multi-time cumulative cost Function (13), representing the cumulative reward within the triggering interval, is dependent on the evolving system state $x_{t}$, showcasing pronounced temporal dependency. To effectively capture temporal dependencies, reduce sensitivity to data fluctuations, and boost accuracy, we employ RNNs for the approximation of the cost Function (13). The approximation involves modeling the total cost when a control action ${\bar{u}}_{l}$ is taken under state $x_{t}$ and maintained over the triggering interval $\tau_{t}$. We denote this network as the critic network. The RNN-based critic network adeptly navigates the system’s temporal dynamics, offering a robust estimation of the multi-time cumulative cost function across various states and control actions. Assuming a discrete time unit dt and a triggering moment $t_{l}$, the triggering interval $tau_{t}$ is given by $tau_{t}=t_{l+1}-t_{l}=n\cdot dt$, with time step *n* indicating the number of discrete program cycles before updating the control input. The system dynamics at each triggering moment are:

$$x_{k+1}=Ax_{k}+B{\bar{u}}_{l},k\in \left[t_{l},t_{l}+dt,\ldots ,t_{l}+n\cdot dt\right].$$

Let the input sequence for RNNs be $v_{k}=\left[x_{k},{\bar{u}}_{l},n\cdot dt\right]$, then the multi-time cumulative cost function fitting process is represented as:   

(19) $$\begin{array}{c} h_{k+1}=\tanh\left(W_{xh}v_{k}+W_{hh}h_{k}+b_{h}\right)\\ y_{k+1}=W_{hy}h_{k+1}+b_{y}\\ G\left(x_{t},{\bar{u}}_{l},\tau_{t}\right)\approx G\left(x_{t},{\bar{u}}_{l},\tau_{t}|W\right)=\left(y_{k}|k=t_{l}+n\cdot dt\right), \end{array}$$

while $W_{xh},W_{hh},W_{hy}$ denote the parameter matrices. $G\left(x_{t},{\bar{u}}_{l},\tau_{t}\right)$ represents the true reward function based on the self-triggered policy, used to evaluate the expected total return by executing the control input ${\bar{u}}_{l}$ continuously within the triggering interval $\tau_{t}$ from the initial state $x_{t}$. $G\left(x_{t},{\bar{u}}_{l},\tau_{t}|W\right)$ is the neural network approximation parameterized by *W*.

Then, given that the control and triggering strategies are solely dependent on the current state of the system, employing a multilayer perceptron (MLP) for their approximation is a suitable approach. These strategies are effectively parameterized using a dual-actor network structure.

(20) $$\left\{\begin{array}{c} \tau_{t}=H\left(x_{t}\right)\approx H_{\theta }\left(x_{t}\right)\\ {\bar{u}}_{l}=U\left(x_{t},\tau_{t}\right)\approx U_{\mu }\left(x_{t},\tau_{t}\right) \end{array}\right.$$

where parameters θ and μ are updated using the policy gradient method. This update process takes into account the estimated multi-time cumulative cost function value and the evaluation of the triggering and control policies. The critic network guides the actor network in generating actions that optimize both control performance and triggering strategy, all while maintaining stability constraints. This process unfolds in two stages: policy evaluation and policy update. During the policy evaluation stage, the current policy’s effectiveness is assessed using evaluation metrics, and a critic network is trained to provide an accurate policy assessment. In the subsequent policy update stage, the gradients of the policy parameters are calculated with respect to the evaluation functions. Gradient descent is then employed to optimize both policies.

For evaluating a self-triggered policy, training RNNs to approximate the state-value function is effective. This training process minimizes the prediction error, which is the difference between the actual return and the RNN’s prediction. The process is articulated through the following loss function:

(21) $$\begin{array}{c} L\left(W\right):=\frac{1}{n}\sum_{i=1}^{n}{\left(G\left({x_{t}}_{i},{{\bar{u}}_{l}}_{i},{\tau_{t}}_{i}|W\right)-\sum_{t=t_{l}}^{t_{l}+{\tau_{t}}_{i}}\left({x_{t}}_{i}^{T}Q{x_{t}}_{i}+{{\bar{u}}_{l}}_{i}^{T}R{\bar{u}}_{li}\right)\right)}^{2}. \end{array}$$

**Remark** **3.** *We can observe that the form of the cost function is represented as the cumulative sum of single-step costs within a given trigger time interval. Given the unknown system model, it becomes impossible to derive an analytical function relating the cost values to the trigger time interval, control actions, and states. Consequently, we cannot compute the gradients of the policy parameters with respect to J and η based on the cost function through automatic differentiation. Therefore, we resort to approximating the cost function using neural networks. This approach enables us to capture the analytical relationship of cost values with respect to the trigger time interval, control actions, and states. By leveraging the output values of the neural network, we can calculate the gradients of the policy parameters relative to J and η, thereby guiding policy optimization.*

The objective is to minimize this error to obtain the optimal parameters $W^{*}$ for the critic network. We utilize the Adam optimizer in TensorFlow for this purpose, achieving the objective through backpropagation of error:

(22) $$\begin{array}{c} W^{*}=\underset{W}{\arg\min}\;L\left(W\right). \end{array}$$

Once the critic network is trained and the multi-time cumulative cost function is approximated, the policy evaluation process assesses the control and trigger policies based on the learned multi-time cumulative cost function. This involves using the critic network to calculate the control performance index *J* and the triggering strategy evaluation metric η for a given initial state and policy:  

(23) $$\left\{\begin{array}{c} J={\left(G\left(x_{t},{\bar{u}}_{l},\tau_{t}|W\right)+V_{\beta }\left(x_{\tau_{t}}\right)-V_{\beta }\left(x_{0}\right)\right)}^{2}\\ \eta =G\left(x_{t},{\bar{u}}_{l},\tau_{t}|W\right)+V_{\beta }\left(x_{\tau_{t}}\right) \end{array}\right.$$

These evaluation indices are utilized to update the parameters of the actor network through the policy gradient method, aiming to find the optimal control and trigger policies that minimize the total cost.

In the policy update phase, the parameters of the actor network, θ and μ, are updated by calculating the gradients of the evaluation indices with respect to the triggering and control policies. The policies are then optimized through gradient descent in the direction that minimizes the evaluation indices until convergence. The updated equations are as follows:

(24) $$\left\{\begin{array}{c} \theta_{i+1}=\theta_{i}-\alpha \nabla_{\tau_{t}}J\nabla_{\theta }H_{\theta }\left(x_{t}\right)\\ \mu_{i+1}=\mu_{i}-\alpha \nabla_{{\bar{u}}_{l}}\eta \nabla_{\mu }U_{\mu }\left(x_{t},\tau_{t}\right) \end{array}\right.$$

Thus, the critic network calculates the multi-time cumulative cost value and provides the policy gradient to continuously optimize the control and trigger policies. In each iteration *i*, alternating between RNN-based policy evaluation and policy improvement steps enables the gradual enhancement of the policy for better performance. The self-triggered control based on hierarchical reinforcement learning is outlined in Algorithm 1.
**Algorithm 1** Model-free self-triggered control based on HRL.**Initialize** W,θ,β**for** ep=0,1,…,N **do**    $t\leftarrow 0,x_{0}\leftarrow random\left(x\right)$    **for** $t\leq t_{final}$ **do**        Choose ${\bar{u}}_{l}$ and $\tau_{t}$ using actor network with noisy signal        Apply ${\bar{u}}_{l}$ during $\tau_{t}$ and read $x_{t}$,$r_{t}$ on each sampling time unit dt        Add ($x_{t}$,${\bar{u}}_{l}$,n·dt,$r_{t}$) to the experience replay buffer D        **if** buffersize≥mini-batchsize **then**           **for** data in buffer **do**               Compute $L\left(W\right):=\frac{1}{n}\sum_{i=1}^{n}{\left(G\left(x_{ti},{\bar{u}}_{li},\tau_{ti}|W\right)-\sum_{t=t_{l}}^{t_{l}+\tau_{ti}}\left(x_{t_{i}}^{T}Qx_{t_{i}}+{\bar{u}}_{l_{i}}^{T}R{\bar{u}}_{li}\right)\right)}^{2}$               Using the adam optimizer to update the critic network parameters *W*               Compute the performance evaluation metrics *J* and η               Update the actor network parameters θ and μ using the formula (24)           **end for**        **end if**    **end for****end for**

## 5. Improving Neural Network Initialization Sensitivity Issues with Pre-Training Methods

In pre-training methodologies, selecting an appropriate generic dataset is crucial. Under situations involving data-driven self-triggered control based on HRL, networks are generally employed to learn and approximate the dynamic behavior of the system and to develop control laws that ensure the system’s performance and stability. If prior knowledge about system dynamics is available, it can be used to select generic labels. For example, known dynamic characteristics of the system can be utilized as these labels.

In NMCS, we need to use HRL training for different subsystems. After one of the subsystems is trained, we can use the actor network of the self-triggered controller that has been trained successfully in this subsystem as prior knowledge. In the subsequent training process of other subsystems’ self-triggered controllers, we pre-train the initial actor network so that it learns part of the knowledge of the previous successful strategy. Then, in the subsequent reinforcement learning stage, we fine-tune the network parameters to make them adapt to the new system.

Assuming that we have a trained actor network, $U_{\lambda }\left(x_{t},\tau_{t}\right)$ and $H_{\sigma }\left(x_{t}\right)$, where λ and σ are the network parameters. Our goal is to use this strategy as a form of prior knowledge for the network to learn from, thereby ensuring that the initial control strategy network possesses certain convergence characteristics as it enters the formal reinforcement learning phase. This approach helps to avoid difficulties in network parameter convergence or training failures during subsequent learning due to reliance on a sub-optimal strategy.

Initially, under different initial conditions, we collect sequences of states,

$$x_{t}={\left[x_{0},x_{1},\ldots ,x_{n}\right]}^{T},$$

and input them into the triggering actor network $H_{\sigma }\left(x_{t}\right)$ to obtain the timing interval sequence,

$$\tau_{t}={\left[\tau_{0},\tau_{1},\ldots ,\tau_{n}\right]}^{T}.$$

Let $v_{t}=\left[x_{t},\tau_{t}\right]$ serves as the input sequence for the neural network.

Inputting $v_{t}$ into the control actor network $U_{\lambda }\left(x_{t},\tau_{t}\right)$ yields the network output vector

$$u_{t}={\left[u_{0},u_{1},\ldots ,u_{n}\right]}^{T}.$$

The training dataset can thus be obtained as:

$$D=\{\left(v_{1},u_{1}\right),\left(v_{2},u_{2}\right),\ldots ,\left(v_{n},u_{n}\right)\}.$$

These data are used for pre-training within the MLP aimed at fitting the control strategy ${\hat{u}}_{t}=U_{\eta }\left(x_{t},\tau_{t}\right)$. The loss function is defined as:

(25) $$L\left(\mu \right)=\frac{1}{n}\sum_{i=1}^{n}{\left({\hat{u}}_{ti}-{u_{t}}_{i}\right)}^{2}.$$

Therefore, the updated formula for the control strategy network becomes:

(26) $$\mu_{i+1}=\mu_{i}-\alpha \nabla_{\mu }L\left(\mu \right).$$

By using gradient descent, the control strategy network approximates the trained networks $U_{\lambda }\left(x_{t},\tau_{t}\right)$, ensuring that the control actions *u* generated based on *x* stabilize the system. This narrows down the strategy exploration space in reinforcement learning, thereby accelerating the learning process and reducing the probability of training failure. The pre-training method of the control actor network is outlined in Algorithm 2.
**Algorithm 2** Pre-training method for control actor network.**Initialize** μ,K**for** ep=0,1,…,N **do**    $t\leftarrow 0,x_{0}\leftarrow random\left(x\right)$    **for** $t\leq t_{final}$ **do**        Choose $\tau_{t}=H_{\sigma }\left(x_{t}\right)$        Choose $u_{t}=U_{\lambda }\left(x_{t},\tau_{t}\right)$        Apply $u_{t}$ after the sampling time unit $\tau_{t}$        Add ($x_{t}$,$\tau_{t}$,$u_{t}$) to the data buffer D        **if** buffersize≥mini-batchsize **then**           **for** data in D **do**               Generated by the control strategy network ${\hat{u}}_{t}=U_{\eta }\left(x_{t},\tau_{t}\right)$               Compute $L\left(\mu \right)=\frac{1}{n}\sum_{i=1}^{n}{\left({\hat{u}}_{ti}-u_{ti}\right)}^{2}$               Using the adam optimizer to update the network parameters μ           **end for**        **end if**    **end for****end for**

## 6. Simulation Result

In this section, we delve into a thorough examination of the hierarchical reinforcement learning self-triggered control (HRLSTC) algorithm’s performance, utilizing the mathematical model of the brushed DC motor as our test subsystem. This examination is critical to assess the algorithm’s effectiveness, particularly focusing on the impacts of pre-training methods and the use of critic networks based on RNNs. We begin by presenting the continuous-time dynamics of the brushed DC motor, represented by the following expression:

$$\dot{x}=\left[\begin{array}{cc} -\frac{R}{L} & -\frac{k_{e}}{L}\\ \frac{k_{t}}{J} & -\frac{B_{v}}{J} \end{array}\right]x+\left[\begin{array}{c} \frac{1}{L}\\ 0 \end{array}\right]u$$

Here, the parameters R=0.4Ω, L=0.8H, $K_{t}=6.3\;\mathrm{Nm}/A$, $K_{e}=4.3\;V/\mathrm{rpm}$, $J=0.08\;\mathrm{kg}\cdot m^{2}$, $B_{v}=0.001\;N\cdot m$ are detailed with their respective values and the state vector $x={\left[i_{a}\left(t\right),\omega \left(t\right)\right]}^{⊤}$, encapsulating the armature current $i_{a}$ and the angular velocity ω. To enable accurate simulations suitable for digital implementation, we convert the continuous model into its discrete counterpart, selecting a sampling period of dt=0.01(s). This transposition yields a discrete-time state space equation as follows:

$$\begin{array}{cc} & x_{k+1}=\left[\begin{array}{cc} 0.973 & -0.053\\ 0.779 & 0.978 \end{array}\right]x_{k}+\left[\begin{array}{c} 0.012\\ 0.004 \end{array}\right]u_{k}\\ & =:A_{d}x_{k}+B_{d}u_{k}. \end{array}$$

Subsequently, we define the discrete-time cost function (8) and the Lyapunov function (where $V_{\beta }\left(x\right)=\beta x^{T}Px$) as follows. These functions are integral to evaluating the performance and stability of the HRLSTC algorithm:

$$Q=\left[\begin{array}{cc} 1 & 0\\ 0 & 1 \end{array}\right],\;R=0.01,\;P=\left[\begin{array}{cc} 4.772 & 2.199\\ 2.199 & 2.931 \end{array}\right].$$

**Figure 6:**
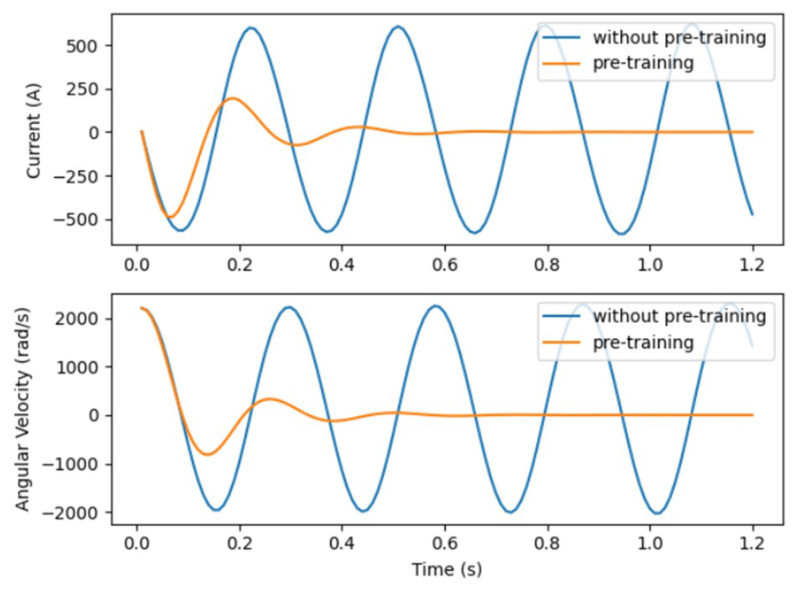
State responses under pre-training policy.

With the preliminary setup in place, we embark on pre-training the control strategy network via Algorithm 2. This phase only requires a few minutes, which is negligible compared to the time spent in the formal reinforcement learning process. It is markedly evident that, absent pre-training, the control strategy is futile in converging the system states (as depicted in Figure 6). However, post pre-training, the network garners invaluable experience, enabling system state convergence without the intervention of the triggering network. Despite the non-optimality of the current control strategy, the foundation laid by the pre-training significantly expedites the fine-tuning process through HRL, leading to a swifter convergence towards the desired strategy as opposed to a non-pre-trained model.

**Figure 7:**
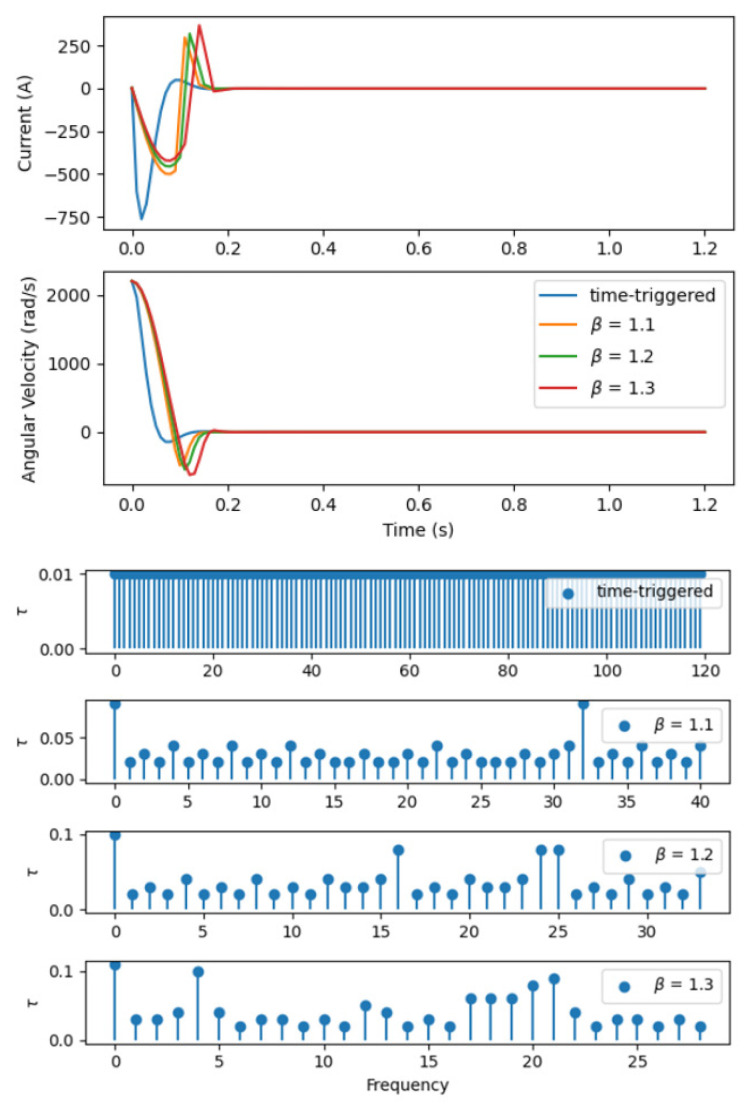
State responses under HRLSTC.

Upon completing the pre-training phase, we proceed with Algorithm 1, initiating the system at state $x_{0}=\left[5,2200\right]$, and setting an appropriate learning rate for the process, specifically $10^{-3}$ for the critic network and $10^{-5}$ for both two actor networks. Our simulation, spanning 250 episodes, each lasting from 0 to 1.2 s, allows us to gather comprehensive response data, which are then systematically incorporated into the experience replay buffer. This methodical approach is designed to capture a wide array of system behaviors under different conditions. Empirical results, as depicted in Figure 7, clearly demonstrate that under periodic control, the system exhibits a notably rapid convergence rate. In contrast, when the system operates under HRLSTC, it achieves a control effect comparable to periodic control but significantly reduces the frequency of control updates, thereby saving computational resources. Moreover, by adjusting the size of parameter β, an intricate balance between control performance and computational resource consumption is achieved in the context of HRLSTC for NMCS.

**Figure 8:**
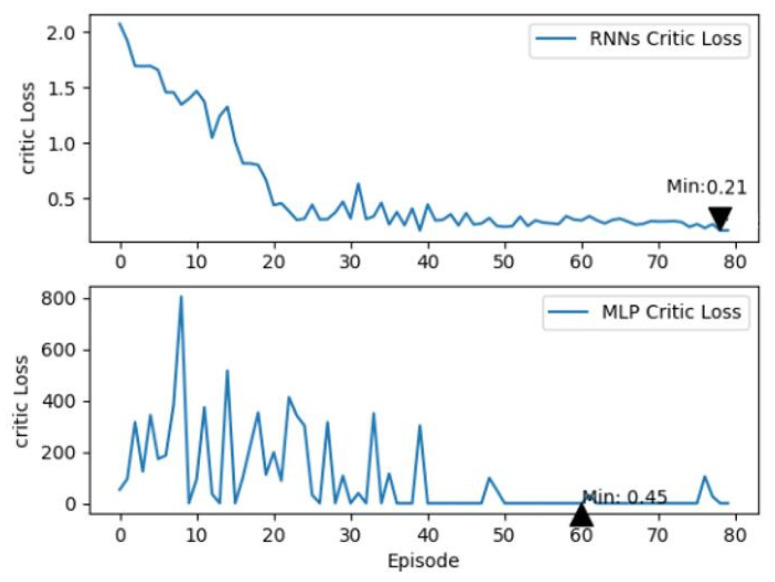
Performance between RNNs and MLP.

Additionally, a comparative study was conducted to evaluate the fitting performance of RNNs versus MLPs, as depicted in Figure 8. This figure clearly presents the outcomes of our analysis, offering a visual representation of the performance disparities between these two network types. It is noteworthy that, when provided with an equal number of training batches, the MLP displays significant error fluctuations in the initial phase of training, indicating instability. This trend continues until it approaches convergence after approximately 40 episodes, highlighting the challenges in achieving stability with MLP during the early training stages. In contrast, the RNNs display a markedly smoother loss trajectory, achieving convergence after just 20 episodes. This contrast highlights the relative robustness and efficiency of RNNs in this specific learning context. Notably, the minimum loss recorded for RNNs is 0.21, significantly lower than the MLP’s 0.45. This substantial difference underlines the superior fitting accuracy of RNNs in our simulation, suggesting their potential advantage for similar control tasks. This underscores the prowess of RNNs in adeptly capturing the time-dependent relationship inherent in the multi-time cumulative cost function, thereby accelerating the critic network’s learning trajectory and ameliorating fitting accuracy.

**Figure 9:**
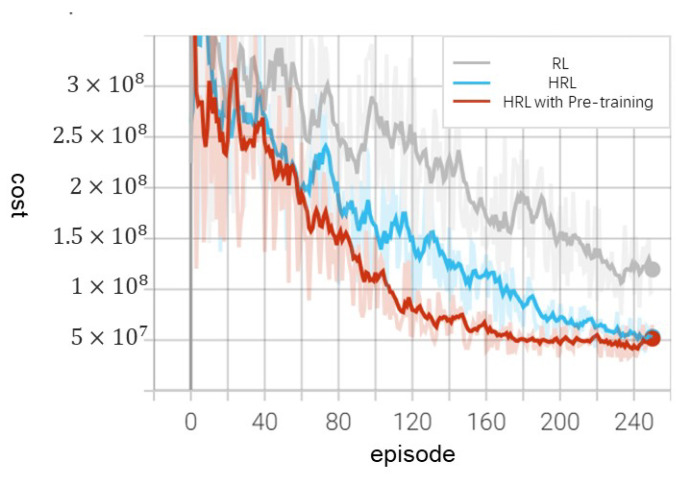
Episode costs under different policy.

In conclusion, Figure 9 effectively illustrates the substantial impact of the pre-training methodology on the learning efficacy of the algorithm. This is evidenced by the enhanced speed of convergence in terms of episode costs when compared to the model lacking pre-training. This finding underscores the critical role of pre-training in optimizing the performance of HRLSTC algorithms. This notable improvement is primarily due to the pre-training method, which imparts crucial prior knowledge to the control strategy network. It aids in understanding the generic features vital for optimal control and self-triggered mechanisms. As a result, this approach circumvents the inefficiency of continuous trial-and-error processes inherent in sub-optimal strategies, facilitating a faster convergence towards the most effective self-triggered control strategy. On the other hand, Figure 9 also highlights the improvements in learning efficiency of the model-free self-triggered controller based on reinforcement learning, as investigated by previous researchers. We contrast our approach with methods lacking a hierarchical structure. Unlike these methods, our HRLSTC demonstrates enhanced learning efficiency while maintaining a consistent environment, learning rate, and initial state. From the figure, it is evident that the convergence speed of episode costs is faster when employing HRLSTC compared to the RL-based method. This suggests that our approach can more rapidly learn the policy that minimizes the total reward.

## 7. Conclusions

This research introduces a pioneering model-free, self-triggered control framework for reducing computational burdens in NMCS. It segments the self-triggered control strategy into higher and lower layers. The higher layer guides the lower layer in decision-making, effectively reducing the exploration space of the lower-level policy and enhancing learning efficiency. Subsequently, the strategic use of RNNs enables the critic network to adeptly capture the temporal dynamics inherent in the learning phase, significantly improving the fitting efficiency and precision of the multi-time cumulative cost function. Additionally, we have developed a pre-training methodology that equips the control strategy network with initial parameters infused with prior knowledge, further boosting reinforcement learning’s efficiency.

Simulation results demonstrate that our model-free self-triggered controller, powered by HRL, effectively develops control and triggering policies that ensure system stability. By adjusting the scaling factor β, a finely tuned balance between performance and computational cost is achieved, underscoring the algorithm’s effectiveness. The employment of RNNs for precise multi-time cumulative cost function approximation, combined with the pre-training method, not only accelerates the learning process but also significantly enhances training efficiency, showcasing the innovation of our framework.

## Data Availability

The data that support the findings of this study are available from the corresponding author upon reasonable request.
